# Parameterization and optimization of the menthol force field for molecular dynamics simulations

**DOI:** 10.1007/s00894-016-3082-1

**Published:** 2016-09-07

**Authors:** Mateusz Jasik, Borys Szefczyk

**Affiliations:** Advanced Materials Engineering and Modelling Group, Faculty of Chemistry, Wrocław University of Technology, Wybrzeże Wyspiańskiego 27, 50-370 Wrocław, Poland

**Keywords:** Menthol, OPLS-AA, Force field parameterization, GROMACS

## Abstract

Menthol’s various biological properties render it a useful component for medical and cosmetological applications, while its three centers of asymmetry mean that it can be used in a range of organic reactions. Menthol-substituted ionic liquids (ILs) have been found to exhibit promising antimicrobial and antielectrostatic properties, as well as being useful in organic catalysis and biochemical studies. However, so far, a force field designed and validated specifically for the menthol molecule has not been constructed. In the present work, the validation and optimization of force field parameters with regard to the ability to reproduce the macroscopic properties of menthol is presented. The set of optimized potentials for liquid simulations all atom (OPLS-AA) compatible parameters was tested and carefully tuned. The refinement of parameters included fitting of partial atomic charges, optimization of Lennard-Jones parameters, and recalculation of the dihedral angle parameters needed to reproduce quantum energy profiles. To validate the force field, a variety of physicochemical properties were calculated for liquid menthol. Both thermodynamic and kinetic properties were taken into account, including density, surface tension, enthalpy of vaporization, and shear viscosity. The obtained force field was proven to accurately reproduce the properties of the investigated compound while being fully compatible with the OPLS-AA force field.

**Electronic supplementary material:**

The online version of this article (doi:10.1007/s00894-016-3082-1) contains supplementary material, which is available to authorized users.

## Introduction

Menthol [1-methyl-4-(1-methylethyl) cyclohexan-3-ol] is a naturally occurring product, commonly obtained from the oil of plants of the *Mentha* genus. Its various biological and biochemical properties make it useful for medical and cosmetological applications such as antiseptics, food preservatives and as a flavouring, as well as a therapeutic agent in pain and irritation treatments [1, 2]. In chemistry, its three centers of asymmetry make menthol a desirable precursor in organic synthesis. Considering also its non-toxicity, reasonable price and environmental-friendliness, menthol is used as a starting molecule in a vast number of reactions, the products of which are used in many applications. The use of menthol in organic chemistry, as well as possible applications of its derivatives, has been thoroughly reviewed elsewhere [3]. Recently, menthol has been used as a substituent in the production of several imidazolium-based ionic liquids (ILs) that present promising antimicrobial and antielectrostatic properties [4–6].

Both menthol and menthol-substituted ILs have been the subject of several studies employing computational chemistry methods—either molecular dynamics (MD) or quantum chemistry [7–13]. However, to the authors’ best knowledge, none of the earlier works has developed a force field designed and validated specifically for the menthol molecule. All previous MD simulations of menthol or menthol derivatives (according to data provided by authors) were conducted with default parameters (for the respective force field), usually prepared in general for hydrocarbons or alcohols. However, especially if ILs are to be simulated, the necessity for a carefully prepared force field for a given molecule must be underlined [14]. The goal of this work was therefore to examine the aptitude of existing parameters in performing MD simulations of pure menthol and furthermore to check the possibility of optimizing the potential or finding a different set of parameters with better performance. The desired force field should fulfill the following requirements: (1) it should reproduce the physicochemical properties of the investigated substances, such as density, surface tension, enthalpy of vaporization or shear viscosity, with good agreement with experimental values, and (2) the force field should be compatible with the optimized potentials for liquid simulations all atom (OPLS-AA) force field [15, 16] that is commonly used to simulate molecular liquids.

## Methods

### Parameterization strategy

The aim of this work was to obtain the best possible force field for menthol compounds that would be able to reproduce their properties in a reliable way, maintaining compatibility with the OPLS-AA standard. The general strategy was to create sets of force field parameters for the menthol molecule, to test them by calculating selected physicochemical properties of liquid menthol, and, based on these calculations, to optimize the parameters to improve the accuracy of the results. The whole process was based on repeating those three operations—obtaining new parameters, calculating (recalculating) chosen properties, and tuning a part or the whole set of parameters. The variables that were changed across sets of used parameters were ε and σ from the Lennard-Jones (LJ) potential, partial atomic charges and Fourier coefficients for dihedral angles describing rotation of menthol’s isopropyl and hydroxyl group, while other bonding terms were taken from the original OPLS-AA force field [15, 16].

### Reoptimization of chosen dihedral angle parameters

The Fourier coefficients for dihedral angles were optimized for both the isopropyl and hydroxyl groups to reproduce the energy profiles calculated using quantum mechanical methods. During this procedure, the respective angle was stepped by 10° in the 0–360° range. The RHF/6-31G(d) energy was calculated for each conformer (while keeping the rest of the molecule fixed). The Fourier coefficients were optimized to minimize the difference between the energy calculated at Hartree-Fock level and the energy defined by force field equations:

1 $$ {\displaystyle {\sum}_a\left(\varDelta {E}_{ff}(a)-\varDelta {E}_{QM}(a)\right)}= \min $$

where α = 0, 10,20 ..., 360; *E*_QM_ is the energy calculated at RHF level and the *E*_ff_ is the sum of intramolecular terms—Coulomb and van der Waals intramolecular interaction and the optimized dihedral term.

### Molecular systems used

The initial molecular system for MD simulations was constructed using 219 menthol molecules, inserted into a box at random positions. The box dimensions were 3.947 × 3.947 × 3.947 nm (after equilibration of the system). Periodic boundary conditions were applied in all simulations. When calculating the surface tension of the system, the box was extended along the *z* axis, so that its final length in the *z* dimension was three times bigger than in *x*/*y* (12 nm). When calculating the enthalpy of vaporization, a single menthol molecule was inserted into the original, 64 nm^3^ box. When calculating the shear viscosity of liquid menthol, the created system contained 876 menthol molecules inserted into a box extended along the *z* axis (final box dimension, after equilibration of the system: 3.996 nm × 3.996  nm× 16.382 nm). For all simulations, the original OPLS-AA force field was used [15, 16], along with the parameters being optimized. Geometry of the system was initially optimized using the steepest-descent method, until all forces were below 200 kJ mol^−1^ nm^−2^. The short-range Coulomb interactions, as well as short-range van der Waals interactions, were calculated within 1.0 nm cut-offs. The simulations in NPT ensemble were preceded by a preliminary equilibration run using Berendsen’s thermostat and barostat [17] that lasted for 1 ns. For the rest of the simulations (both in NVT and NPT ensembles), the Nosé-Hoover thermostat [18, 19] was used. For the production run in the NPT ensemble, the Parrinello-Rahman barostat was applied [20]. All simulations were performed with a time step of 1 fs, with all bonds constrained using the LINCS algorithm [21]. A simulation time of 10 ns or 100 ns was used, depending on the calculated property (as described in “Calculations of physicochemical properties”). Uncertainties of calculated properties were estimated by dividing the production run into 200 ps blocks, and calculating averages and their standard deviations (SD) for these blocks. For the calculations, which were based on ten times longer MD simulations, the uncertainties were estimated using 2000 ps blocks. These standard deviations are presented in this work as a measure of uncertainty of the calculated properties. The division of the simulations into parallel processes was performed by a threading procedure available by default in the GROMACS program [22].

### Calculations of physicochemical properties

Density (ρ) at different temperatures was obtained by averaging the results from 10 ns NPT simulations. Pressure in these simulations was set at 1 bar. The results were compared with experimentally obtained densities at different temperatures [23, 24]. The first two points (293 K and 298 K) required special treatment in order to improve sampling of the configuration space: first a 10 ns NPT simulation at elevated temperature (473 K) was performed. Next, ten evenly spaced snapshots were selected from the trajectory. These snapshots were used to initialize simulations at low temperature, and the density was averaged over the ten simulations.

Surface tension (γ) was calculated from an NVT ensemble, 100 ns simulation of a system with the box *z* dimension extended to approximately three times the original length (box size: 4.026 nm × 4.026 nm × 12.189 nm). In this case, the periodic images of simulated liquid were separated by about 8 nm vacuum in this dimension. After equilibration, the components of the pressure tensor were collected. The surface tension was estimated using the formula [25]

2 $$ \gamma =0.5\cdot {\mathrm{L}}_{\mathrm{z}}\left(\left\langle {\mathrm{P}}_{\mathrm{z}\mathrm{z}}\right\rangle -\frac{\left\langle {\mathrm{P}}_{\mathrm{xx}}\right\rangle -\left\langle {\mathrm{P}}_{\mathrm{yy}}\right\rangle }{2}\right) $$

The factor 0.5 is due to the two interfaces present in the system. The results were compared with experimentally obtained surface tension values at different temperatures [23].

Enthalpy of vaporization (Δ*H*_vap_) was calculated based on an approach that assumes that the sum of vibrational and kinetic energies is equal for the gas and liquid phase [26]. The formula for enthalpy of vaporization is therefore written as:

3 $$ \varDelta {\mathrm{H}}_{\mathrm{vap}}=\mathrm{E}\left(\mathrm{g}\right)-\mathrm{E}\left(\mathrm{l}\right)+\mathrm{R}\mathrm{T} $$

where:

E(g) = : E_dih_(g) + E_intra_(g)
E(l) = : E_dih_(l) + E_intra_(l) + E_inter_(l)

*E*_dih_ describes the dihedral energy term, *E*_intra_ is the nonbonding intramolecular interaction energy, and *E*_inter_ is the intermolecular interaction energy. Terms (g) and (l) refer to the gas and liquid phase, respectively. The gas phase was simulated in a 100 ns NVT simulation of a single menthol molecule, while the liquid phase was represented as a 10 ns NVT simulation of a 219-molecule system. The results were compared with experimentally obtained enthalpies of vaporization at different temperatures [27].

Shear viscosity (η) was calculated using non-equilibrium periodic perturbation method [28]. A 10 ns NVT simulation has been performed in a rectangular box with *z* dimension extended (box size: 3.996 nm × 3.996 nm × 16.382 nm). A periodic acceleration a_x_(z) was applied along the *x*-axis

4 $$ {a}_x(z)=A\; \cos \left(\frac{2\pi }{L_z}z\right) $$

where *L*_z_ is the length of the box in the *z* dimension and *A* is an arbitrary amplitude parameter. The *A* parameter has been chosen carefully, to provide the best possible statistics while not moving the system too far from the equilibrium [28]. The simulations were performed for a range of *A* values (0.004, 0.005, 0.006, 0.01, 0.015, 0.02, 0.03, 0.04, 0.05, 0.06, 0.07 nm/ps^2^). To achieve optimal results, the value of *A* = 0.005 nm/ps^2^ was chosen for calculating final viscosity, which was computed according to the formula:

5 $$ \upeta =\frac{{\mathrm{A}}_{\uprho}}{\mathrm{V}}{\left(\frac{{\mathrm{L}}_{\mathrm{z}}}{2\uppi}\right)}^2 $$

where *V* is defined as:

$$ \mathrm{V}\left(\mathrm{t}\right)=\frac{2{\displaystyle {\sum}_{\mathrm{i}=1}^{\mathrm{N}\;\mathrm{atoms}}{\mathrm{m}}_{\mathrm{i}}{\mathrm{v}}_{\mathrm{i},\mathrm{x}}\left(\mathrm{t}\right) \cos \left({\mathrm{kr}}_{\mathrm{i},\mathrm{z}}\left(\mathrm{t}\right)\right)}}{{\displaystyle {\sum}_{\mathrm{i}=1}^{\mathrm{N}\;\mathrm{atoms}}{\mathrm{m}}_{\mathrm{i}}}} $$

The results were compared with experimentally obtained shear viscosity of liquid menthol at different temperatures [29].

## Results and discussion

It is worth emphasizing that the parameterization strategy and procedure described in “Methods” and discussed here is considered to be standard for acquiring OPLS-AA force field parameters [16]. The rationale behind it is theoretically justified and, importantly, has been proved recently to provide good results for developing imidazolium-based IL force fields [30]. The key feature is its ability to accurately reproduce the physicochemical properties of the studied compound. These properties were therefore used as a basic mean to evaluate the constructed force field.

**Fig. 1:**
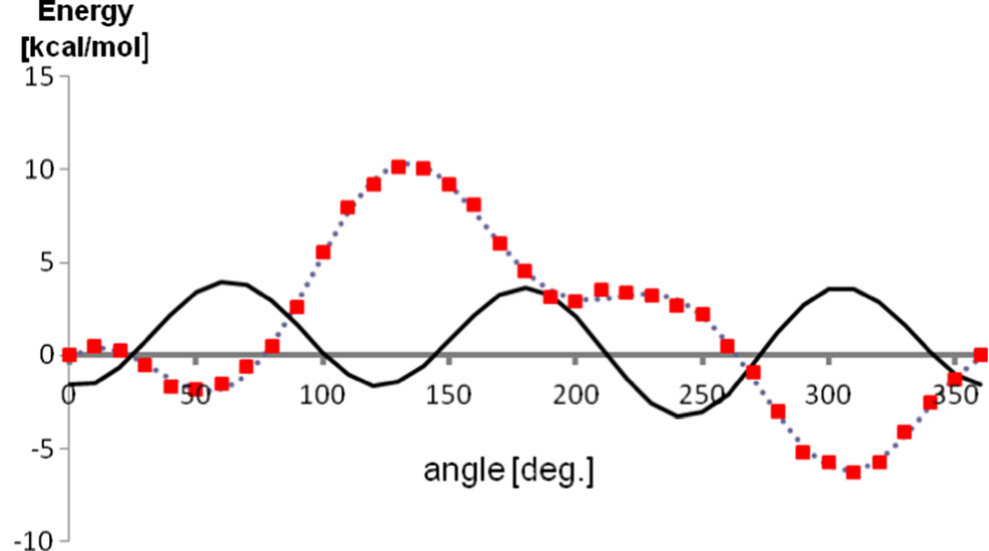
Profile of the dihedral angle describing the rotation of the hydroxyl group plotted using optimized parameters. Quantum chemical energy is shown using squares, optimized force field energy is shown using dashed line, force field energy calculated with original (non-optimized) parameters is shown using solid line

**Fig. 2:**
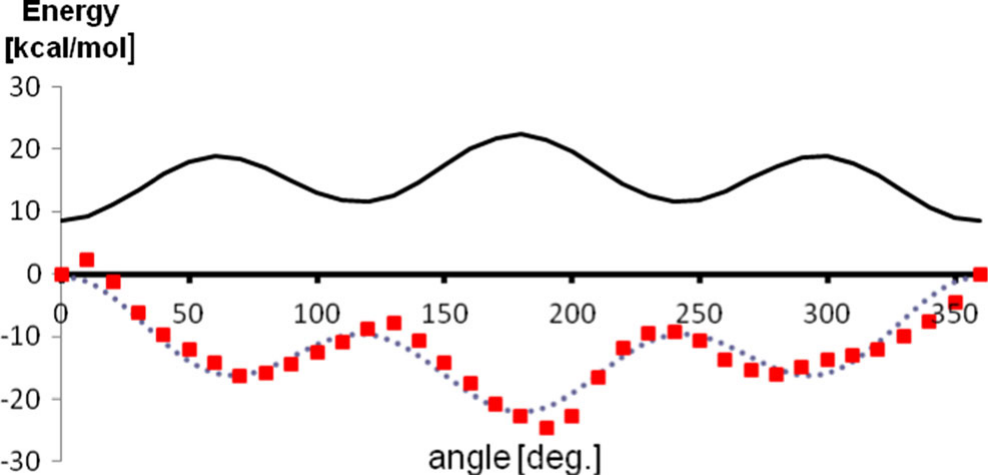
Profile of the dihedral angle describing rotation of isopropyl group plotted using optimized parameters. Quantum chemical energy is shown using squares, optimized force field energy is shown using dashed line, force field energy calculated with original (non-optimized) parameters is shown using solid line.

The bonded parameters—bond and angle interactions, as well as most of the dihedral angle interactions—were therefore taken from the original OPLS-AA force field as they do not contribute much change to those properties. For the dihedral angle interactions, default parameters were also used, with the exception of the angles describing rotation of the hydroxyl and isopropyl group of menthol, which are the only truly labile and asymmetric groups in the molecule. After optimization of the remaining parameters, these angles were tuned to fit the energy profile from restricted Hartree-Fock (RHF) calculations, using the procedure described in “Methods”. The energy profiles of the reoptimized angles, as well as those described by original OPLS-AA parameter, are shown in Figs. 1 and 2. These figures show that the tuned dihedral parameters perform much better in describing accurate energetic profiles, making a significant qualitative difference. The whole set of bonded parameters used is shown in Table 1.

**Table 1:** Force field parameters for menthol molecule

Force field parameter	Value			
Lennard-Jones parameters [15, 16]				
Atom type	σ [Å]		ε [kJ mol^−1^]	
CT^a^	3.50		0.327921	
HC^a^	2.50		0.149055	
O	3.12		0.844645	
HO^a^	0.00		0.000000	
Bond stretching parameters [15, 16]				
Bond type	*r*_eq_ [Å]		*K*_r_ [10^−3^ kJ mol^−1^ nm^−2^]	
CT–CT	1.529		224.2624	
CT–HC	1.090		284.5120	
CT–O	1.410		267.7760	
O–HO	0.945		462.7504	
Angle bending parameters [15, 16]				
Angle type	θ_eq_ [^o^]		*K*_θ_ [kJ mol^−1^ rad^-2^]	
HC–CT–HC	107.8		276.144	
HC–CT–CT	110.7		313.800	
CT–CT–CT	112.7		488.273	
CT–CT–O	109.5		418.400	
CT–O–HO	108.5		460.240	
HC–CT–O	109.5		292.880	
Torsional parameters [kJ/mol]				
Dihedral type	V_1_	V_2_	V_3_	V_4_
CT–CT–CT–CT [16]	−3.3472	−0.20920	0.8368	0.0
CT–CT–CT–HC [16]	−7.531	0.000	−1.255	0.0
HC–CT–CT–HC [16]	−7.531	0.000	−1.255	0.0
CT–CT–CT–O^b^	7.159	−2.092	2.774	0.0
HC–CT–CT–O^b^	−0.00002	0.000	1.958	0.0
HC–CT–O–HO^b^	0.000	0.000	0.000	0.0
CT–CT–O–HO^b^	7.448	−1.430	7.261	0.0
Torsional parameters for isopropyl group				
CT–CT–CT–CT^b^	12.681	−1.557	2.934	0.0
CT–CT–CT–HC^b^	0.000	0.000	0.000	0.0
HC–CT–CT–HC^b^	0.000	0.000	0.000	0.0

^a^Symbols used: *CT* sp^3^ carbon (all carbons in molecule), *HC* hydrogen attached to a carbon, *HO* hydrogen of hydroxyl group, *O* oxygen of hydroxyl group

^b^Obtained in this work

Special attention has been given to the parameters describing intermolecular interactions, that is the van der Waals parameters and partial atomic charges. The scaling factors for 1–4 Coulomb and van der Waals interactions were conserved from the original force field (amounting to 0.5), the same applies to the exclusion of 1–2 and 1–3 nonbonded interactions. To assess whether or not the van der Waals parameters from the OPLS-AA force field are able to estimate the forces between the molecules correctly, both ε and σ parameters describing the depth of the energy profile and the zero-energy interatomic distance, respectively, were tuned. Both smaller and larger values of these parameters were tested, the change being applied by scaling the ε and/or σ values for all atoms in the menthol molecule. The original values of van der Waals parameters, proposed in the OPLS-AA force field [15, 16], while sufficient for describing the static properties of liquid menthol, had to be adjusted for the calculation of transport properties, namely the shear viscosity of menthol. The depolarization of atomic charges (described below), which was necessary to properly describe the shear viscosity, caused the rest of the calculated properties to deviate from the experimental values. It was found that increasing the ε value on all atoms up to 118.75 % of the original values was optimal to account for the undesirable changes. The final values of nonbonding parameters used are presented in Table 1. Simultaneously to the optimization of van der Waals parameters, atomic charges were optimized and re-optimized. To obtain the initial set of partial atomic charges, the geometry of menthol molecule was optimized at the HF/6-31G* level of theory. After that, a single point calculation was performed at the MP2 level, using the aug-cc-pVTZ basis set, with the f-type function excluded, which is a standard procedure for the OPLS-AA force field [16]. The initial charges were calculated according to the CHELPG algorithm [31]. The charges were then rounded up, symmetrized and assigned to atoms. As those charges did not reproduce the properties of menthol (particularly the surface tension of pure menthol) to a satisfactory degree, other sets of charges were prepared and tested using several different methods. These other sets of charges that were tested originated from various computational methods, i.e., NPA [32], AIM [33], and Löwdin [34, 35] methods, as well as the charges proposed by the authors of OPLS-AA force field [15, 16]. Sets obtained via the NPA method, as well as those proposed by OPLS-AA force field, were additionally tested with increased or decreased van der Waals parameters. The final set of charges comprised the charges proposed by the authors of the OPLS-AA force field, which were obtained using method analogous to described above, but applied to 36 organic liquids [15, 16]. Those charges were assigned to all atoms with the exception of the hydroxyl group of menthol. For this group, charges obtained from the NPA method [32] were used, resulting in charges more polarized than those from the original force field (−0.72 charge on the oxygen atom and 0.45 on the hydrogen atom, before the depolarization described below). These charges were adjusted slightly to keep the molecule neutral. Calculations of the charges using the NPA method were conducted by a method analogous to the one described above—optimization of menthol geometry at the HF/6-31G* level, followed by a single point calculation at the MP2 level, using the aug-cc-pVTZ(−f) basis set. None of the combinations of charges described above gave better agreement with experimental values than the set chosen for the final force field. It was also found that, to describe the shear viscosity of liquid menthol correctly, charges from the OPLS-AA field needed to be depolarized, by scaling them down to 85 % of their original value. Such a procedure is commonly used when performing IL MD simulations, to account for polarization and charge transfer effects. The reduction of the formal charge (typically by 10 %, 20 % or even 30 %) significantly improves the transport properties of IL [14, 36–39]. This issue has been thoroughly studied elsewhere, including ab initio molecular dynamics simulations and quantum chemical calulations [37, 40, 41]. It was found that employing the described scheme for liquid menthol also substantially improves the dynamic properties of the simulated system. The final atomic charges of menthol molecule are presented in Table 2.

**Table 2:**
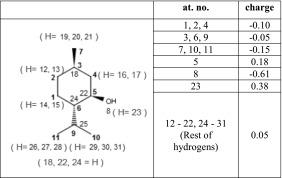
Atomic charges set used in the force field for a menthol molecule

The summary of all calculated properties is presented in Table 3 and compared with their experimentally measured counterparts. Table 4 provides a comparison of chosen properties calculated with standard OPLS-AA parameters [15, 16] with the same properties calculated using the optimized parameters presented in this work.

**Table 3:** Comparison of experimental and calculated properties of liquid menthol for the obtained force field.

		Experimental	Calculated	SD^a^	Deviation from experimental value^b^
		Density [kg m^−3^]			[%]
T [K]	293	890 [24]	921.1	1.1	3.49
	298	923.6 [24]	917.3	1.2	0.68
	318	889 [23]	902.0	3.4	1.46
	353	877 [23]	872.1	3.5	0.56
	393	865.1 [23]	836.4	3.8	3.32
		Surface tension [mN m^−1^]			[%]
T [K]	313	31.61 [23]	28.6	8.2	9.52
	333	29.2 [23]	27.1	6.5	7.19
	353	27.82 [23]	24.9	5.4	10.50
	373	25.62 [23]	22.7	3.6	11.40
	393	23.75 [23]	20.6	3.0	13.26
		Enthalpy of vaporization [kJ mol^−1^]			[%]
T [K]	382	56.92 [27]	52.8	5.7	7.24
	421	53.42 [27]	51.1	4.8	4.34
	473	48.42 [27]	49.5	5.5	2.23
		Shear viscosity [mPa s^−1^]			[%]
T [K]	320	10.8 [29]	5.9	1.2	45.37
	350	2.3 [29]	3.8	0.58	65.22
	380	0.83 [29]	2.8	0.36	237.4

^a^Standard deviation of the calculated values

^b^Percentage deviation of the results calculated with obtained force field from the corresponding experimental values

**Table 4:** Density and enthalpy of vaporization of liquid menthol calculated using standard and optimized parameters, in comparison with experimental values

		Experimental	Standard parameters	Relative error [%]	Optimized parameters	Relative error [%]
Density [kg m^−3^]						
T [K]	293	890 [24]	896.1	0.7	921.1	3.5
	318	889 [23]	875.0	1.6	902.0	3.4
	393	865.1 [23]	801.8	7.3	836.4	3.8
Enthalpy of vaporization [kJ mol^−1^]						
T [K]	382	56.92 [27]	31.9	44.0	52.8	7.2
	421	53.42 [27]	29.7	44.4	51.1	4.3
	473	48.42 [27]	26.7	44.9	49.5	2.2

**Fig. 3:**
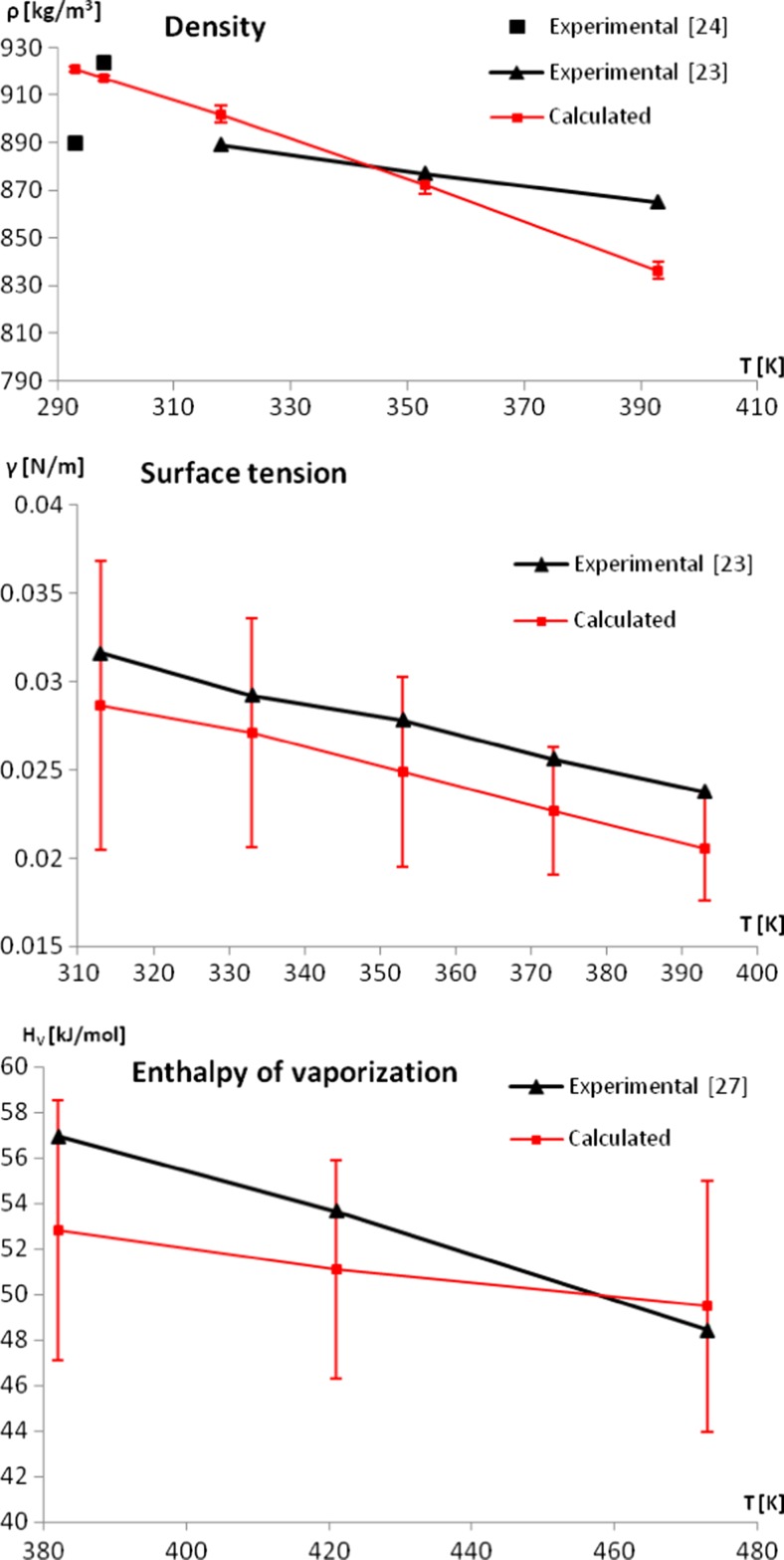
Comparison between calculated and experimental [23, 24, 27] thermodynamic properties of pure menthol

**Fig. 4:**
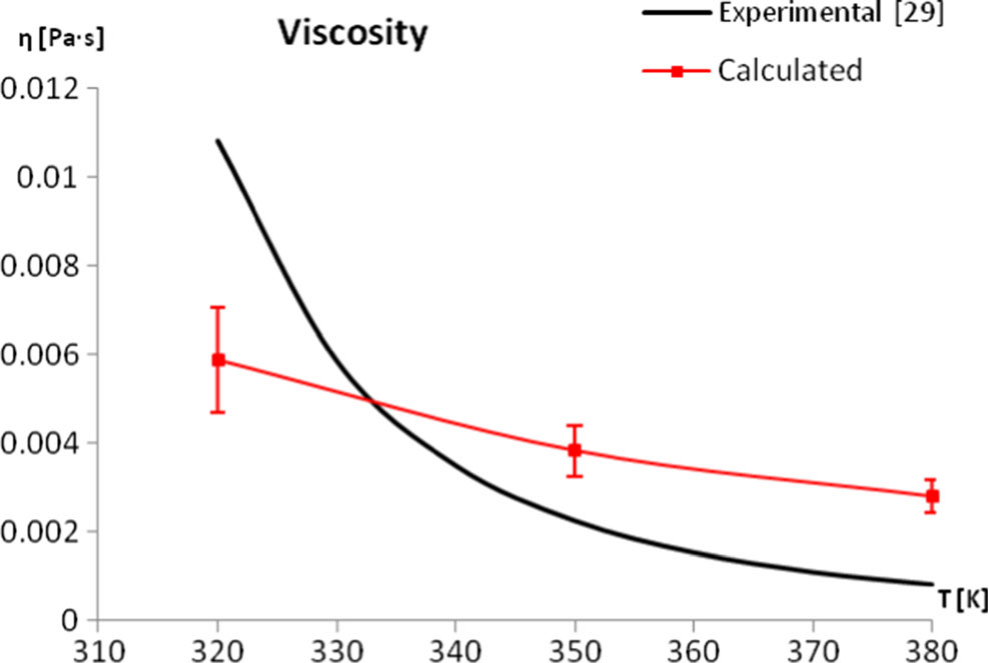
Comparison between calculated and experimental [29] shear viscosity of pure menthol. The experimental value is represented as a calculated function within the recommended temperature range [29]

The obtained potential allowed the density of pure menthol to be calculated with good accuracy, although the calculated density displays a stronger temperature dependence than the results found experimentally. As described below, this is different from other calculated properties, which tend to show a weaker temperature dependency than the experimental one. It should be mentioned that a better correlation with experimental values can be obtained by using non-depolarized partial atomic charges (that is, charges without scaling down to the 85 % of their original values), at the cost of shear viscosity, which would be several orders of magnitude too high. Therefore, the final set of parameters has to be considered optimal when taking into account static and dynamic properties. For the density of menthol, the best accordance with experiment occurs within the temperature range from 320 K to 360 K. It should be noted that the available sets of experimental densities for menthol were obtained from two separate sources [23, 24]. It is therefore hard to estimate changes in the experimental density between 293 K and 310 K (see Fig. 3). Calculations of the surface tension of pure menthol turned out to be computationally demanding. In order to avoid the uncertainties of the surface tension values being too high, the simulation time had to be extended to 100 ns (compared to 10 ns simulations for calculation of other properties). This is associated with the character of the calculated property; since the surface tension is obtained based on the components of pressure tensor, it is affected strongly by large oscillations in pressure, which are to be expected. The obtained force field is capable of producing surface tension values with good accordance to experimental values [23]; the values obtained are from 2 × 10^−3^ to 3 × 10^−3^ N m^−1^ lower than the experimental ones (Fig. 3). The calculated values of the enthalpy of vaporization also remain in good accordance with experimental results [27]. While the calculated enthalpies of vaporization stay within the proper range of values, the temperature dependence of this property is weaker than indicated by experiment, which leads to slightly underestimated values at lower temperatures. This property is prone to large uncertainty values, due to the large variation associated with gas phase simulations; the gas phase state is modelled by the simulation of a single menthol molecule. It is therefore recommended to perform longer simulations for this phase (in this work 100 ns simulations were performed). The shear viscosity of a liquid, being a dynamic property, is one of the most important characteristics that should validate the force field, especially for compounds that demand more carefully adjusted parameters [14] . The arbitrarily chosen parameter A (the amplitude of the induced fluctuations) turns out to be of great importance for calculating the viscosity of menthol. Only a narrow range of tested amplitudes allowed to calculate the investigated property reliably. Values of A higher than 0.06 nm/ps^2^ caused the viscosity to decrease rapidly, indicating that the system was too strongly perturbed, and diverged from the metastable state of non-equilibrium dynamics. Simultaneously, the lower amplitudes are not capable of inducing an organized motion in the system (compared to normal thermal motions), the friction is too low, and the uncertainties become too high; decreasing A from 0.06 nm ps^−2^ to 0.04 nm ps^−2^ can increase the calculated standard deviation by as much as 59 % in lower temperatures. Therefore, the final value of A = 0.05  nm ps^−2^ was chosen as a compromise, which resulted in relatively low values of calculated standard deviations and did not disturb the system too much. The obtained force field allows the shear viscosity of liquid menthol to be reproduced well within the range of the experimental values [29] (Fig. 4, Table 3), although it is not able to correctly describe the changes of viscosity in the whole temperature range. The problem could possibly be remedied by including polarization terms in the force field. A very similar unsatisfactory temperature dependence of the calculated viscosity was observed for the MD of liquid water [42], with the addition of polarization terms as the suggested solution. It has also been confirmed that including polarization effects is important for the proper description of the temperature-dependent properties of organic liquids such as ethylene glycol [43]. It was, however, beyond the aim of this study to investigate this solution, as one of our main goals was to keep the force field compatible with the standard of the OPLS-AA force field.

## Conclusions

In this work, a force field for the MD simulations of liquid menthol was constructed, evaluated and optimized. The presented force field is fully compatible with the OPLS-AA force field and can possibly be used together with other potentials adhering to this standard. Furthermore, the proposed potential avoids introducing any non-standard energy terms or scaling factors inconsistent with the OPLS-AA standard. The potential transferability of the developed force field was additionally tested by performing several MD simulations of menthol-substituted, imidazolium-based ILs (unpublished data), resulting in a reliable description of their densities. The potential describing rotation of hydroxyl and isopropyl groups was fitted to the quantum chemical profiles, which significantly improved the torsional potentials. The force field was validated by calculating various physicochemical properties of liquid menthol. Both static (density, surface tension, enthalpy of vaporization) and transport (shear viscosity) properties were calculated. This range of investigated properties should be considered an advantage of the developed force field, as it was aimed at providing versatility and good performance while assessing many different characteristic simultaneously. While several combinations of parameters were tested, the final version of the force field was judged to be optimal, that is, to describe all the aforementioned properties with the best accordance with their respective experimental counterparts. The force field performs well for the chosen properties, although it suffers a bit when temperature dependence is considered. However, the force field should be applied with caution when reproducing transport properties or those based on the gas phase of the investigated compound considering the issues discussed above. It is worth emphasizing that the force field describes the viscosity of menthol with acceptable accuracy; this property is considered to be difficult to reproduce in MD simulations and, as such, is a good measure of the quality of tested parameters.

## Electronic supplementary material

Below is the link to the electronic supplementary material.

Supporting information Topology of menthol molecule and parameter set in GROMACS format. (PDF 21 kb)
