# Supplementary material for: Parameterization and optimization of the menthol force field for molecular dynamics simulations
Source: J Mol Model. 2016 Sep 7;22(10):234. doi: 10.1007/s00894-016-3082-1 (PMC5014899; doi:10.1007/s00894-016-3082-1)
Supplement: Supplementary file 1 — Topology of menthol molecule and parameter set in GROMACS format. (PDF 21 kb) [file 894_2016_3082_MOESM1_ESM.pdf]

Topology in Gromacs format

```
[ moleculetype ]
; name nrexc
MEN        3

[ atoms ]
; nr  type  resnr residue atom  cgnr charge    mass
   1  MCR   1       MEN   C1    6   -0.10    12.011
   2  MCR   1       MEN   C2    7   -0.10    12.011
   3  MC    1       MEN   C3    1   -0.05    12.011
   4  MCR   1       MEN   C4    2   -0.10    12.011
   5  MCO   1       MEN   C5    3    0.18    12.011
   6  MC    1       MEN   C6    4   -0.05    12.011
   7  MCM   1       MEN   C7    1   -0.15    12.011
   8  MO    1       MEN   O8    3   -0.61    15.9994
   9  MC    1       MEN   C9    5   -0.05    12.011
  10  MCM   1       MEN   C10   5   -0.15    12.011
  11  MCM   1       MEN   C11   5   -0.15    12.011
  12  MHR   1       MEN   H12   7    0.05    1.0080
  13  MHR   1       MEN   H13   7    0.05    1.0080
  14  MHR   1       MEN   H14   6    0.05    1.0080
  15  MHR   1       MEN   H15   6    0.05    1.0080
  16  MHR   1       MEN   H16   2    0.05    1.0080
  17  MHR   1       MEN   H17   2    0.05    1.0080
  18  MHC   1       MEN   H18   1    0.05    1.0080
  19  MHM   1       MEN   H19   1    0.05    1.0080
  20  MHM   1       MEN   H20   1    0.05    1.0080
  21  MHM   1       MEN   H21   1    0.05    1.0080
  22  MHH   1       MEN   H22   3    0.05    1.0080
  23  MHO   1       MEN   H23   3    0.38    1.0080
  24  MHC   1       MEN   H24   4    0.05    1.0080
  25  MHC   1       MEN   H25   5    0.05    1.0080
  26  MHM   1       MEN   H26   5    0.05    1.0080
  27  MHM   1       MEN   H27   5    0.05    1.0080
  28  MHM   1       MEN   H28   5    0.05    1.0080
  29  MHM   1       MEN   H29   5    0.05    1.0080
  30  MHM   1       MEN   H30   5    0.05    1.0080
  31  MHM   1       MEN   H31   5    0.05    1.0080

[bonds ]
;      ai  aj  funct
    3   2    1
    3   4    1
    4   5    1
    5   6    1
    6   1    1
```

```
    2   1     1
    2   12    1
    2   13    1
    1   14    1
    1   15    1
    4   16    1
    4   17    1
    3   18    1
    3   7     1
    7   19    1
    7   20    1
    7   21    1
    5   8     1
    5   22    1
    8   23    1
    6   9     1
    6   24    1
    9   25    1
    9   11    1
    9   10    1
    11  26    1
    11  27    1
    11  28    1
    10  29    1
    10  30    1
    10  31    1

[pairs]
;     ai        aj        funct
    19    18    1
    19    2     1
    19    4     1
    20    18    1
    20    2     1
    20    4     1
    21    18    1
    21    2     1
    21    4     1
    7     13    1
    7     12    1
    7     1     1
    7     16    1
    7     17    1
    18    1     1
    18    13    1
    18    12    1
    18    5     1
```

| | | |
|---|---|---|
| 18 | 16 | 1 |
| 18 | 17 | 1 |
| 3 | 14 | 1 |
| 3 | 15 | 1 |
| 3 | 6 | 1 |
| 13 | 4 | 1 |
| 12 | 4 | 1 |
| 1 | 4 | 1 |
| 2 | 16 | 1 |
| 2 | 17 | 1 |
| 2 | 5 | 1 |
| 12 | 14 | 1 |
| 12 | 15 | 1 |
| 12 | 6 | 1 |
| 13 | 14 | 1 |
| 13 | 15 | 1 |
| 13 | 6 | 1 |
| 2 | 5 | 1 |
| 2 | 9 | 1 |
| 2 | 24 | 1 |
| 14 | 5 | 1 |
| 14 | 9 | 1 |
| 14 | 24 | 1 |
| 15 | 5 | 1 |
| 15 | 9 | 1 |
| 15 | 24 | 1 |
| 1 | 8 | 1 |
| 1 | 22 | 1 |
| 1 | 10 | 1 |
| 1 | 11 | 1 |
| 1 | 25 | 1 |
| 24 | 22 | 1 |
| 24 | 4 | 1 |
| 24 | 8 | 1 |
| 24 | 25 | 1 |
| 24 | 10 | 1 |
| 24 | 11 | 1 |
| 9 | 4 | 1 |
| 9 | 22 | 1 |
| 9 | 8 | 1 |
| 25 | 5 | 1 |
| 10 | 5 | 1 |
| 11 | 5 | 1 |
| 6 | 23 | 1 |
| 6 | 17 | 1 |
| 6 | 16 | 1 |
| 6 | 3 | 1 |

```
    6     26   1
    6     27   1
    6     28   1
    6     29   1
    6     30   1
    6     31   1
    23    4    1
    22    23   1
    22    3    1
    22    16   1
    22    17   1
    8     16   1
    8     17   1
    8     3    1
    5     7    1
    25    26   1
    25    27   1
    25    28   1
    25    29   1
    25    30   1
    25    31   1
    26    10   1
    27    10   1
    28    10   1
    29    11   1
    30    11   1
    31    11   1

[angles]
;    ai   aj    ak   funct
    19   7     20    1
    20   7     21    1
    19   7     21    1
    19   7     3     1
    20   7     3     1
    21   7     3     1
    7    3     2     1
    7    3     4     1
    7    3     18    1
    18   3     2     1
    18   3     4     1
    3    2     12    1
    3    2     13    1
    3    2     1     1
    2    3     4     1
    12   2     13    1
    12   2     1     1
```

```
    13   2    1    1
    2    1    6    1
    2    1    14   1
    2    1    15   1
    14   1    15   1
    14   1    6    1
    15   1    6    1
    1    6    5    1
    1    6    24   1
    1    6    9    1
    24   6    5    1
    24   6    9    1
    9    6    5    1
    6    5    8    1
    6    5    22   1
    6    5    4    1
    6    9    11   1
    6    9    10   1
    6    9    25   1
    5    8    23   1
    8    5    22   1
    22   5    4    1
    8    5    4    1
    5    4    16   1
    5    4    17   1
    5    4    3    1
    16   4    17   1
    16   4    3    1
    17   4    3    1
    25   9    11   1
    25   9    10   1
    9    11   26   1
    9    11   27   1
    9    11   28   1
    9    10   29   1
    9    10   30   1
    9    10   31   1
    11   9    10   1
    26   11   27   1
    27   11   28   1
    26   11   28   1
    29   10   30   1
    30   10   31   1
    29   10   31   1

[dihedrals]
;    ai    aj    ak    al    funct
```

| | | | | |
|---|---|---|---|---|
| 19 | 7 | 3 | 18 | 3 |
| 19 | 7 | 3 | 2 | 3 |
| 19 | 7 | 3 | 4 | 3 |
| 20 | 7 | 3 | 18 | 3 |
| 20 | 7 | 3 | 2 | 3 |
| 20 | 7 | 3 | 4 | 3 |
| 21 | 7 | 3 | 18 | 3 |
| 21 | 7 | 3 | 2 | 3 |
| 21 | 7 | 3 | 4 | 3 |
| 7 | 3 | 2 | 13 | 3 |
| 7 | 3 | 2 | 12 | 3 |
| 7 | 3 | 2 | 1 | 3 |
| 7 | 3 | 4 | 16 | 3 |
| 7 | 3 | 4 | 17 | 3 |
| 18 | 3 | 2 | 1 | 3 |
| 18 | 3 | 2 | 13 | 3 |
| 18 | 3 | 2 | 12 | 3 |
| 18 | 3 | 4 | 5 | 3 |
| 18 | 3 | 4 | 16 | 3 |
| 18 | 3 | 4 | 17 | 3 |
| 3 | 2 | 1 | 14 | 3 |
| 3 | 2 | 1 | 15 | 3 |
| 3 | 2 | 1 | 6 | 3 |
| 13 | 2 | 3 | 4 | 3 |
| 12 | 2 | 3 | 4 | 3 |
| 1 | 2 | 3 | 4 | 3 |
| 2 | 3 | 4 | 16 | 3 |
| 2 | 3 | 4 | 17 | 3 |
| 2 | 3 | 4 | 5 | 3 |
| 12 | 2 | 1 | 14 | 3 |
| 12 | 2 | 1 | 15 | 3 |
| 12 | 2 | 1 | 6 | 3 |
| 13 | 2 | 1 | 14 | 3 |
| 13 | 2 | 1 | 15 | 3 |
| 13 | 2 | 1 | 6 | 3 |
| 2 | 1 | 6 | 5 | 3 |
| 2 | 1 | 6 | 9 | 3 |
| 2 | 1 | 6 | 24 | 3 |
| 14 | 1 | 6 | 5 | 3 |
| 14 | 1 | 6 | 9 | 3 |
| 14 | 1 | 6 | 24 | 3 |
| 15 | 1 | 6 | 5 | 3 |
| 15 | 1 | 6 | 9 | 3 |
| 15 | 1 | 6 | 24 | 3 |
| 1 | 6 | 5 | 4 | 3 |
| 1 | 6 | 5 | 8 | 3 |
| 1 | 6 | 5 | 22 | 3 |

| | | | | |
|---|---|---|---|---|
| 1 | 6 | 9 | 10 | 3 |
| 1 | 6 | 9 | 11 | 3 |
| 1 | 6 | 9 | 25 | 3 |
| 24 | 6 | 5 | 22 | 3 |
| 24 | 6 | 5 | 4 | 3 |
| 24 | 6 | 5 | 8 | 3 |
| 24 | 6 | 9 | 25 | 3 |
| 24 | 6 | 9 | 10 | 3 |
| 24 | 6 | 9 | 11 | 3 |
| 9 | 6 | 5 | 4 | 3 |
| 9 | 6 | 5 | 22 | 3 |
| 9 | 6 | 5 | 8 | 3 |
| 25 | 9 | 6 | 5 | 3 |
| 10 | 9 | 6 | 5 | 3 |
| 11 | 9 | 6 | 5 | 3 |
| 6 | 5 | 8 | 23 | 3 |
| 6 | 5 | 4 | 17 | 3 |
| 6 | 5 | 4 | 16 | 3 |
| 6 | 5 | 4 | 3 | 3 |
| 6 | 9 | 11 | 26 | 3 |
| 6 | 9 | 11 | 27 | 3 |
| 6 | 9 | 11 | 28 | 3 |
| 6 | 9 | 10 | 29 | 3 |
| 6 | 9 | 10 | 30 | 3 |
| 6 | 9 | 10 | 31 | 3 |
| 23 | 8 | 5 | 4 | 3 |
| 22 | 5 | 8 | 23 | 3 |
| 22 | 5 | 4 | 3 | 3 |
| 22 | 5 | 4 | 16 | 3 |
| 22 | 5 | 4 | 17 | 3 |
| 8 | 5 | 4 | 16 | 3 |
| 8 | 5 | 4 | 17 | 3 |
| 8 | 5 | 4 | 3 | 3 |
| 5 | 4 | 3 | 7 | 3 |
| 25 | 9 | 11 | 26 | 3 |
| 25 | 9 | 11 | 27 | 3 |
| 25 | 9 | 11 | 28 | 3 |
| 25 | 9 | 10 | 29 | 3 |
| 25 | 9 | 10 | 30 | 3 |
| 25 | 9 | 10 | 31 | 3 |
| 26 | 11 | 9 | 10 | 3 |
| 27 | 11 | 9 | 10 | 3 |
| 28 | 11 | 9 | 10 | 3 |
| 29 | 10 | 9 | 11 | 3 |
| 30 | 10 | 9 | 11 | 3 |
| 31 | 10 | 9 | 11 | 3 |

Parameters in Gromacs format

```
#define _FF_OPLSAA

[ defaults ]
;    nbfunc    comb-rule    gen-pairs    fudgeLJ    fudgeQQ
    1    2         yes         0.5    0.5

[atomtypes]
;  name   bond_type mass    charge  ptype    sigma    epsilon
    MCR  MCR  6    12.011   -0.12  A    3.50000e-01  3.27921e-01
    MC    MC  6    12.011   -0.06  A    3.50000e-01  3.27921e-01
    MCO  MCO  6    12.011    0.21  A    3.50000e-01  3.27921e-01
    MCM  MCM  6    12.011   -0.18  A    3.50000e-01  3.27921e-01
    MO    MO 16    15.9994  -0.72  A    3.12000e-01  8.44645e-01
    MHO  MHO  1    1.0080    0.45  A    0.00000e+00  0.00000e+00
    MHR  MHR  1    1.0080    0.06  A    2.50000e-01  1.49055e-01
    MHM  MHM  1    1.0080    0.06  A    2.50000e-01  1.49055e-01
    MHC  MHC  1    1.0080    0.06  A    2.50000e-01  1.49055e-01
    MHH  MHH  1    1.0080    0.06  A    2.50000e-01  1.49055e-01

[bondtypes]
;    i    j    f    b0    kb
    MCR     MCR   1    0.15290    224262.4
    MCR     MC    1    0.15290    224262.4
    MCR     MCO   1    0.15290    224262.4
    MCO     MC    1    0.15290    224262.4
    MC      MCM   1    0.15290    224262.4
    MC      MC    1    0.15290    224262.4
    MCO     MO    1    0.14100    267776.0
    MCR     MHR   1    0.10900    284512.0
    MC      MHC   1    0.10900    284512.0
    MCM     MHM   1    0.10900    284512.0
    MCO     MHH   1    0.10900    284512.0
    MO      MHO   1    0.09450    462750.4

[angletypes]
;    i    j    k    func    th0    cth
    MHM   MCM   MHM   1    107.800    276.144
    MHM   MCM   MC    1    110.700    313.800
    MCM   MC    MCR   1    112.700    488.273
    MCM   MC    MHC   1    110.700    313.800
    MHC   MC    MCR   1    110.700    313.800
    MC    MCR   MHR   1    110.700    313.800
    MC    MCR   MCR   1    112.700    488.273
    MCR   MC    MCR   1    112.700    488.273
    MHR   MCR   MHR   1    107.800    276.144
```

```
   MHR   MCR   MCR   1    110.700    313.800
   MCR   MC    MCO   1    112.700    488.273
   MCR   MC    MHC   1    110.700    313.800
   MCR   MC    MC    1    112.700    488.273
   MHC   MC    MCO   1    110.700    313.800
   MHC   MC    MC    1    110.700    313.800
   MC    MC    MCO   1    112.700    488.273
   MC    MCO   MO    1    109.500    418.400
   MC    MCO   MHH   1    110.700    313.800
   MC    MCO   MCR   1    112.700    488.273
   MC    MC    MCM   1    112.700    488.273
   MCO   MO    MHO   1    108.500    460.240
   MO    MCO   MHH   1    109.500    292.880
   MHH   MCO   MCR   1    110.700    313.800
   MO    MCO   MCR   1    109.500    418.400
   MCO   MCR   MHR   1    110.700    313.800
   MCO   MCR   MC    1    112.700    488.273
   MHC   MC    MCM   1    110.700    313.800
   MCM   MC    MCM   1    112.700    488.273

[dihedraltypes]
; i    j    k    l    func    c0    c1    c2    c3    c4    c5
 MHM   MCM   MC    MHC   3    0.62760    1.88289   0.0000    -2.5104     0.0   0.0
 MHM   MCM   MC    MCR   3    0.62760    1.88289   0.0000    -2.5104     0.0   0.0
 MCM   MC    MCR   MHR   3    0.62760    1.88289   0.0000    -2.5104     0.0   0.0
 MHC   MC    MCR   MCR   3    0.62760    1.88289   0.0000    -2.5104     0.0   0.0
 MHC   MC    MCR   MHR   3    0.62760    1.88289   0.0000    -2.5104     0.0   0.0
 MHC   MC    MCR   MCO   3    0.62760    1.88289   0.0000    -2.5104     0.0   0.0
 MC    MCR   MCR   MHR   3    0.62760    1.88289   0.0000    -2.5104     0.0   0.0
 MCR   MC    MCR   MHR   3    0.62760    1.88289   0.0000    -2.5104     0.0   0.0
 MHR   MCR   MC    MCR   3    0.62760    1.88289   0.0000    -2.5104     0.0   0.0
 MC    MCR   MCR   MC    3    2.92880   -1.46440   0.20920   -1.67360    0.0   0.0
 MCR   MCR   MC    MCR   3    2.92880   -1.46440   0.20920   -1.67360    0.0   0.0
 MCR   MC    MCR   MCO   3    2.92880   -1.46440   0.20920   -1.67360    0.0   0.0
 MHR   MCR   MCR   MHR   3    0.62760    1.88289   0.0000    -2.5104     0.0   0.0
 MCR   MCR   MC    MCO   3    2.92880   -1.46440   0.20920   -1.67360    0.0   0.0
 MCR   MCR   MC    MC    3    2.92880   -1.46440   0.20920   -1.67360    0.0   0.0
 MHR   MCR   MC    MCO   3    0.62760    1.88289   0.0000    -2.5104     0.0   0.0
 MHR   MCR   MC    MC    3    0.62760    1.88289   0.0000    -2.5104     0.0   0.0
 MCR   MC    MCO   MCR   3    2.92880   -1.46440   0.20920   -1.67360    0.0   0.0
 MCR   MC    MCO   MO    3    2.87441    0.58158   2.09200   -5.54799    0.0   0.0
 MCR   MC    MCO   MHH   3    0.62760    1.88289   0.0000    -2.5104     0.0   0.0
 MHC   MC    MCO   MCR   3    0.62760    1.88289   0.0000    -2.5104     0.0   0.0
 MCR   MC    MC    MCM   3   -3.83272   -1.93967   1.55701   -5.86783    0.0   0.0
 MCR   MC    MC    MHC   3    0.0000     0.0000    0.0000     0.0000     0.0   0.0
 MC    MC    MCO   MCR   3    2.92880   -1.46440   0.20920   -1.67360    0.0   0.0
 MC    MCO   MCR   MHR   3    0.62760    1.88289   0.0000    -2.5104     0.0   0.0
```

| | | | | | | | | | | |
|---|---|---|---|---|---|---|---|---|---|---|
| MHC | MC | MCO | MHH | 3 | 0.62760 | 1.88289 | 0.0000 | -2.5104 | 0.0 | 0.0 |
| MHC | MC | MCO | MO | 3 | 0.97905 | 2.93716 | 0.0000 | -3.9162 | 0.0 | 0.0 |
| MC | MC | MCO | MHH | 3 | 0.62760 | 1.88289 | 0.0000 | -2.5104 | 0.0 | 0.0 |
| MC | MC | MCO | MO | 3 | 2.87441 | 0.58158 | 2.09200 | -5.54799 | 0.0 | 0.0 |
| MHC | MC | MC | MCO | 3 | 0.0000 | 0.0000 | 0.0000 | 0.0000 | 0.0 | 0.0 |
| MCM | MC | MC | MCO | 3 | -3.83272 | -1.93967 | 1.55701 | -5.86783 | 0.0 | 0.0 |
| MC | MCO | MO | MHO | 3 | -0.06119 | 7.16709 | 1.43002 | -14.52161 | 0.0 | 0.0 |
| MC | MCO | MCR | MC | 3 | 2.92880 | -1.46440 | 0.20920 | -1.67360 | 0.0 | 0.0 |
| MC | MC | MCM | MHM | 3 | 0.62760 | 1.88289 | 0.0000 | -2.5104 | 0.0 | 0.0 |
| MHO | MO | MCO | MCR | 3 | -0.06120 | 0.01614 | 1.89092 | 3.65341 | 0.0 | 0.0 |
| MHH | MCO | MO | MHO | 3 | 0.0000 | 0.0000 | 0.0000 | 0.0000 | 0.0 | 0.0 |
| MHH | MCO | MCR | MC | 3 | 0.62760 | 1.88289 | 0.0000 | -2.5104 | 0.0 | 0.0 |
| MHH | MCO | MCR | MHR | 3 | 0.62760 | 1.88289 | 0.0000 | -2.5104 | 0.0 | 0.0 |
| MO | MCO | MCR | MHR | 3 | 0.97905 | 2.93716 | 0.0000 | -3.9162 | 0.0 | 0.0 |
| MO | MCO | MCR | MC | 3 | 2.87441 | 0.58158 | 2.09200 | -5.54799 | 0.0 | 0.0 |
| MCO | MCR | MC | MCM | 3 | 2.92880 | -1.46440 | 0.20920 | -1.67360 | 0.0 | 0.0 |
| MHM | MCM | MC | MCM | 3 | 0.62760 | 1.88289 | 0.0000 | -2.5104 | 0.0 | 0.0 |
| MCM | MC | MCR | MCR | 3 | 2.92880 | -1.46440 | 0.20920 | -1.67360 | 0.0 | 0.0 |
| MHC | MC | MC | MHC | 3 | 0.0000 | 0.0000 | 0.0000 | 0.0000 | 0.0 | 0.0 |
| MHC | MC | MC | MCM | 3 | 0.0000 | 0.0000 | 0.0000 | 0.0000 | 0.0 | 0.0 |
